# Accessibility and socio-economic development of human settlements

**DOI:** 10.1371/journal.pone.0179620

**Published:** 2017-06-21

**Authors:** Samiul Hasan, Xiaoming Wang, Yong Bing Khoo, Greg Foliente

**Affiliations:** 1 Civil, Environmental, and Construction Engineering, University of Central Florida, Orlando, Florida, United States of America; 2 CSIRO Land and Water, Clayton South, Victoria, Australia; 3 Department of Infrastructure Engineering, The University of Melbourne, Parkville, Victoria, Australia; TNO, NETHERLANDS

## Abstract

Access to facilities, services and socio-economic opportunities plays a critical role in the growth and decline of cities and human settlements. Previous attempts to explain changes in socio-economic indicators by differences in accessibility have not been convincing as countries with highly developed transport infrastructure have only seen marginal benefits of infrastructure improvements. Australia offers an ideal case for investigating the effects of accessibility on development since it is seen as home to some of the most liveable cities in the world while, at the same time, it also has some of the most isolated settlements. We investigate herein the connectivity and accessibility of all 1814 human settlements (population centers exceeding 200 persons) in Australia, and how they relate to the socio-economic characteristics of, and opportunities in, each population center. Assuming population as a proxy indicator of available opportunities, we present a simple ranking metric for a settlement using the number of population and the distance required to access all other settlements (and the corresponding opportunities therein). We find a strikingly unequal distribution of access to opportunities in Australia, with a marked prominence of opportunities in capital cities in four of the eight states. The two largest cities of Sydney and Melbourne have a dominant position across all socio-economic indicators, compared to all the other cities. In general, we observe across all the settlements that a decrease in access to opportunities is associated with relatively greater socio-economic disadvantage including increased median age and unemployment rate and decreased median household income. Our methodology can be used to better understand the potential benefits of improved accessibility based on infrastructure development, especially for remote areas and for cities and towns with many socio-economically disadvantaged population.

## Introduction

Accessibility generally describes “the degree to which a product, device, service, or environment is accessible by as many people as possible” [1], or, from the point of view of community residents, their “ability to reach desired goods, services, activities and destinations” [2]. In recent decades, accessibility measures have been used to understand and guide a range of policies and investment decisions to enable regional development and support sustainability goals [3]. In a relative scale, an accessibility measure rates the services that the population of an area can access and the costs it has to pay using transport infrastructure including road, rail, water, and air connecting that area with other areas [4, 5]. In regional areas, the provision of better transport infrastructure is seen as critical to improving its population’s access to various services and opportunities [6–9]. Furthermore, it is considered to provide better access to the locations of input materials, and lead to markets that are more productive and competitive [10].

Remoteness can be defined as the inverse of relative accessibility. The concept of remoteness, and hence accessibility, is an important dimension of policy development in Australia. Access to available services and opportunities is a major issue [11,12] because of the typically long distances people need to travel for accessing government services. To help plan the provision of many of these services, the Australian Bureau of Statistics (ABS) has adopted a remoteness index known as ARIA (Accessibility/Remoteness Index of Australia) [13], which measures the remoteness of a point based on the physical road distance to the nearest urban center.

Lack of adequate infrastructure hinders access to markets [14,15]. In some of these remote places, most infrastructure is funded by businesses (such as those in the resources industry), or by charging users (such as the case of electricity generation). In these cases, businesses determine infrastructure needs based on users’ willingness to pay, which in turn depends on how the users value such services and how they can access the infrastructure. But left to market forces alone, this may lead to a lack of investment in socioeconomically disadvantaged communities in remote areas.

When a government prioritizes the socioeconomic development of a given area, it typically invests to build new transport infrastructure. For instance, to link the dispersed populations and remote businesses of Northern Australia, the Australian Government is establishing a $5 billion infrastructure plan to provide loans for the construction of major infrastructure (e.g., ports, electricity and water supply, rail, pipelines etc.), with an additional $600 million for constructing new roads [16]. However, the relative benefits or value of different investments is difficult to determine and prioritizing projects accordingly is a big challenge. For example, it is not always straightforward to allocate project benefits that spill across borders, capture the diverse needs from regions, assess the impacts of projects, and satisfactorily address the potential of overstating the benefits and understating the impacts by different stakeholders [16].

Before investing heavily on infrastructure development, the important questions to address are: (a) to what extent the access to services exist in those remote areas related to the more developed regions, and (b) what are the prevailing impacts of such differences?

Australia offers an ideal case for investigating the effects of such inequalities in regional development and accessing opportunities as it has some of the most liveable cities in the world (such as Melbourne, Sydney, Brisbane, Perth and Adelaide) [17], and, at the same time, it has some of the most isolated settlements in the world. Smith [18] reported that desert areas of Australia had population of around 580,000 with a density of 0.11 persons km^-2^ whereas global average for desert area population density was around 24 persons km^-2^.

The role and impact of transport infrastructure in regional development are not clear, and sometimes the direction of causality is questionable [10]. Previous attempts to explain changes in economic indicators (growth and decline) by transport investment or differences in accessibility has been much less successful [19,20]. Countries with highly developed transport infrastructure have seen marginal benefits of infrastructure improvements [21]. Gathering evidence on the impacts of differences in accessibility with a sparsely spread transport infrastructure network is very important.

Understanding the effects of inequality in accessing opportunities in Australia now may have lessons for other places and the world at large in the future. In 2014, 54 per cent of the world’s population was living in cities. The compounding urban agglomeration effects point to a projection that 66 per cent of the world’s population will be living in cities by 2050 [22]. As a result, some settlements in the world could become more remote and socioeconomically disadvantaged compared to the large and fast-growing cities, even though cities will highly depend on these regions for food, water, clean air, waste disposal and recreational activities [18]. The influence of physical accessibility on social and economic development of remote areas need to be better understood.

This paper presents an overall picture of the connectivity and accessibility of all human settlements in Australia, ranked nationally and by state, and relates their accessibility rating to the socio-economic characteristics of each community. This is an important step towards a more explicit or expansive consideration of the influence of improving accessibility and prioritizing transport infrastructure investment into towns and communities that are relatively more socioeconomically disadvantaged than others.

## Methods

### Accessibility measurement

In order to determine the role of transport infrastructure in regional development, we first measure the accessibility of different human settlement centers, referred to as Urban Centre and Localities (UCLs), in Australia (see Data in supplemental information). Accessibility has been a well known concept in regional science and has various formulations [1,12,23–25]. We propose the following formulation to compute the accessibility indicators for different UCLs:

$$A_{i}=\;\Sigma_{L}\Sigma_{j\in L}\;W_{j}exp\left(-\frac{d_{ij}}{{\bar{d}}_{L}}\right);\;L\in \left\{A,\;B,\;C,\;D,\;E,F\right\}$$ (1)

where *A*_*i*_ is the accessibility of region *i*; *L* is the service center category of region *j* to be reached from region *i; W*_*j*_ is the measure of opportunity/activity, here the size of population to be reached in region *j* from region *i; d*_*ij*_ is the distance to be covered to go from *i* to *j*; ${\bar{d}}_{L}$ is the average of the distances from all the areas to their nearest area of service center category *L* (Table 1). The negative exponent of the ratio $\frac{d_{ij}}{{\bar{d}}_{L}}$ is the generalized cost of reaching area *j* from area *i*. It combines two ideas:

1. that the nearby places have greater influence than the remote ones, and
2. that the areas will be treated differently by its service center category and categories with greater average distance generally implies less number of those service centers but having greater influence (typically these categories have greater services and opportunities available, such as major capitals).

**Table 1:** Average distances to service center categories.

Service Center Category	Population	Average Distance to Service Center (km)
A	> = 250,000	362
B	48,000–249,999	256
C	18,000–47,999	192
D	5000–17,999	83
E	1000–4999	33
F	200–999	11

This accessibility index was selected because:

1. We want to reflect the amount of opportunities and services available in different regions which are currently measured by population
2. We want to look at the influence of not only the nearest region but all regions. But since with increasing distance the influence tends to decrease, we use an exponential function to penalize regions with longer distance. In other words, nearby regions have higher influence than the remote ones.
3. We want to differentiate the influence of different types of regions. Hence, we categorize all the regions by the type of service centers and use the mean distance for that type of service center in the exponential function. In particular, we use the ratio between the distance of a region and the mean distance from all the regions to the nearest region of that particular type.

## Results

### Spatial distributions of accessibility

First, we present a general picture of the connectivity of different UCLs with road networks. Figure A in S1 File shows the distribution of human settlements in relation to the major transport network of Australia. In the east coast, there is a high concentration of human settlements particularly in the Brisbane-Sydney-Melbourne (B-S-M) triangle. Most of the UCLs in this region are highly connected with transport infrastructure. The figure also shows the lack of connectivity in Northern Australia. Most small UCLs elsewhere are not connected with the major transport road network, a sharp contrast with the B-S-M triangle.

**Fig 1:**
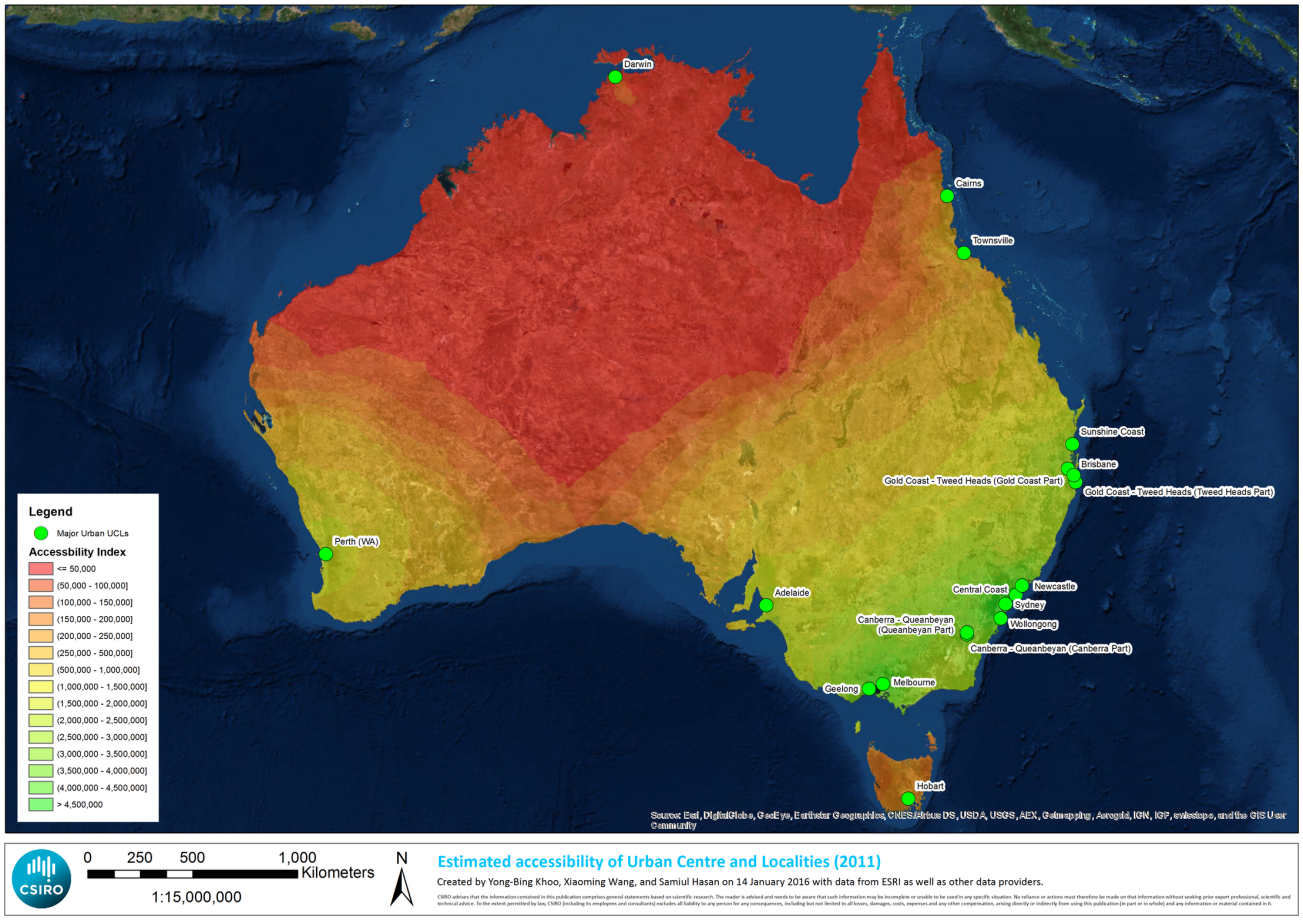
Accessibility values of the UCLs with classification.

The spatial distribution of the accessibility values of the UCLs are shown in Fig 1. The accessibility values were computed using the proposed accessibility index in Eq (1). In general, most UCLs are not highly accessible. Only the cities in the east coast around the B-S-M region have greater accessibility. We see a trend of increasing accessibility values originating from the eastern coast cities. The big cities in the east coast including Brisbane, Sydney, and Melbourne are responsible for the beginning of this trend. A similar pattern is observed for Western Australia with Perth as the origin of the trend. Unlike these regions, there is no increasing pattern observed in Northern Australia in the same scale.

We then create a ranking analysis of the UCLs based on their accessibility values. For ranking purpose, we normalize the accessibility values in a scale from 0 to 100. We consider the highest value for the accessibility index as 100 (in our case, Sydney has the highest accessibility value) and compute the accessibility values of other UCLs as follows:

$$Normalized\;A_{i}=\;\frac{A_{i}}{A_{Sydney}}\times 100$$ (2)

**Fig 2:**
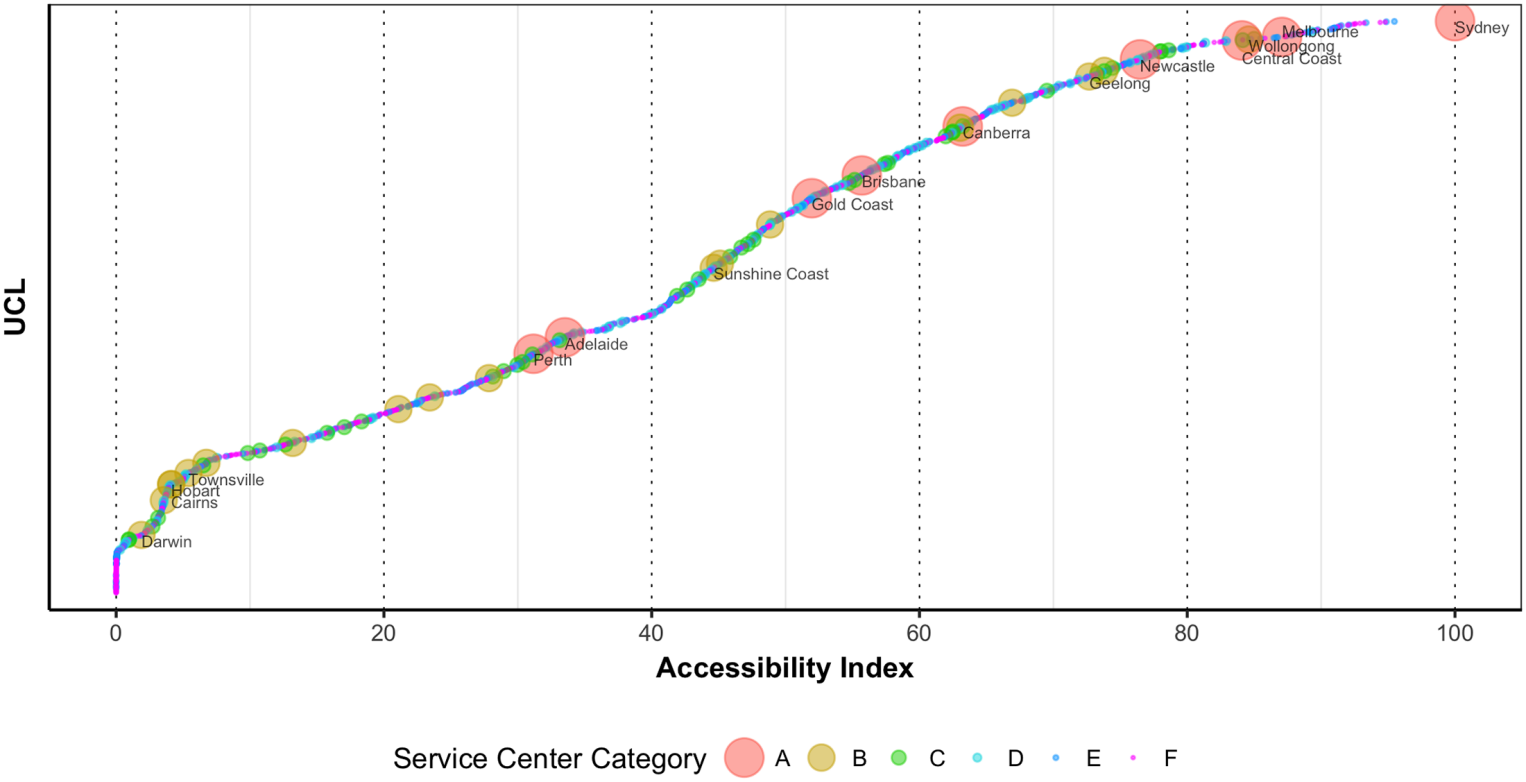
Ranking of UCLs based on accessibility values (Only the UCLs with more than 100,000 populations are labelled with their names).

Fig 2 shows the ranking of the UCLs based on accessibility values. We can see among the cities with greater than 100,000 populations that Sydney has the highest accessibility value and Darwin has the lowest accessibility value. Interestingly, some of the major capital cities such as Adelaide and Perth have lower accessibility values. This ranking clearly shows the unequal distribution of available opportunities across Australia, of which the most concerning one is the domestic opportunity available for Darwin. This could be an inherent characteristic of Australian geography. Although no option might be available to change the ranking, future infrastructure investments can be guided based on this ranking. This is specifically an important consideration for Australia since Darwin is strategically positioned in the Asian region where the advantages of stronger social and economic links are great.

Among different Australian cities, Sydney, Melbourne and the UCLs around them have very high accessibilities (above 80). Newcastle, Geelong, and Canberra have high accessibilities (60 to 80). Brisbane, Gold Coast, Sunshine Coast and the UCLs around them have moderate accessibilities (40 to 60). Adelaide and Perth and the UCLs around them have low accessibilities (20 to 40). Cities such as Townsville, Cairns, Hobart, Darwin and the UCLs around them have very low accessibilities (below 20).

**Fig 3:**
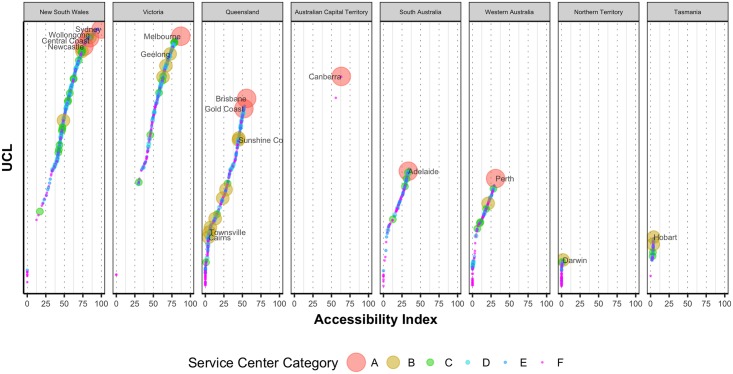
Ranking of UCLs based on accessibility values grouped by state (Only the UCLs with more than 100,000 populations are labelled with their names).

Fig 3 shows the ranking of the UCLs for different states. This confirms the unequal distribution of available opportunities across different states. It is clear that UCLs from South Australia, Western Australia, Northern Territory and Tasmania have the least opportunities available for them compared to other states such as New South Wales, Victoria and Queensland. The hierarchical structure of the accessibility of UCLs in a state starts from the major capital city, as expected. And the accessibility values of the other UCLs in a given state then just follow that of its capital city.

### Socio-economic impacts of accessibility

In order to assess the socio-economic impacts of the unequal access to opportunities across Australia, we correlate different variables including median age, median household income, median rent, median mortgage repayment, unemployment rate and the Socio-Economic Indexes for Areas (SEIFA) of the UCLs with the corresponding accessibility values. SEIFA is developed by the Australian Bureau of Statistics (ABS) to rank areas in Australia according to relative socio-economic advantage and disadvantage [26]. The SEIFA indexes seek to summarize the socio-economic conditions of an area using relevant information from the Census of Population and Housing.

**Fig 4:**
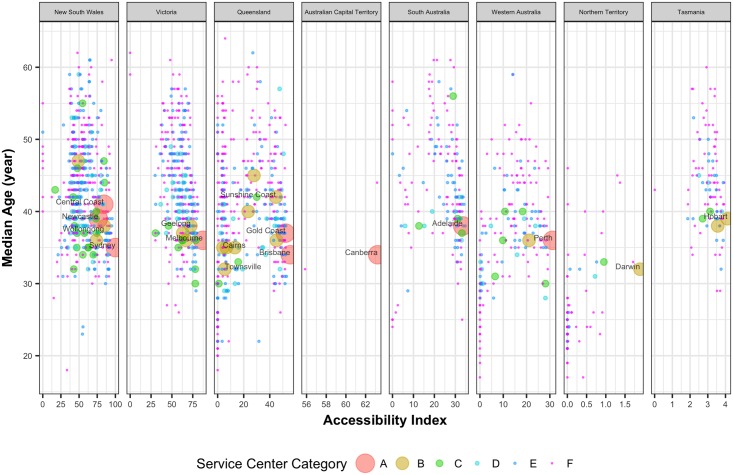
Median age of population against the corresponding normalized accessibility values of the UCLs.

Fig 4 shows the median age of the population living in the UCLs against their corresponding accessibility values. All the major cities have median age between 30 and 40. As one would naturally expect, with more employment opportunities available, more accessible cities attract younger population. On the contrary, other than the major cities, median age is increasing with the decrease of accessibility values. This pattern is consistent across different states. The gap between the minimum values and the maximum values is also increasing with decreasing accessibility values. This is also happening for all the socio-economic indicators. This reflects potentially different liveability levels among the regions with low accessibility values, creating social and economic inequality. Less accessible places having greater median age will be challenging in the future since older people will have to access health care facilities more.

**Fig 5:**
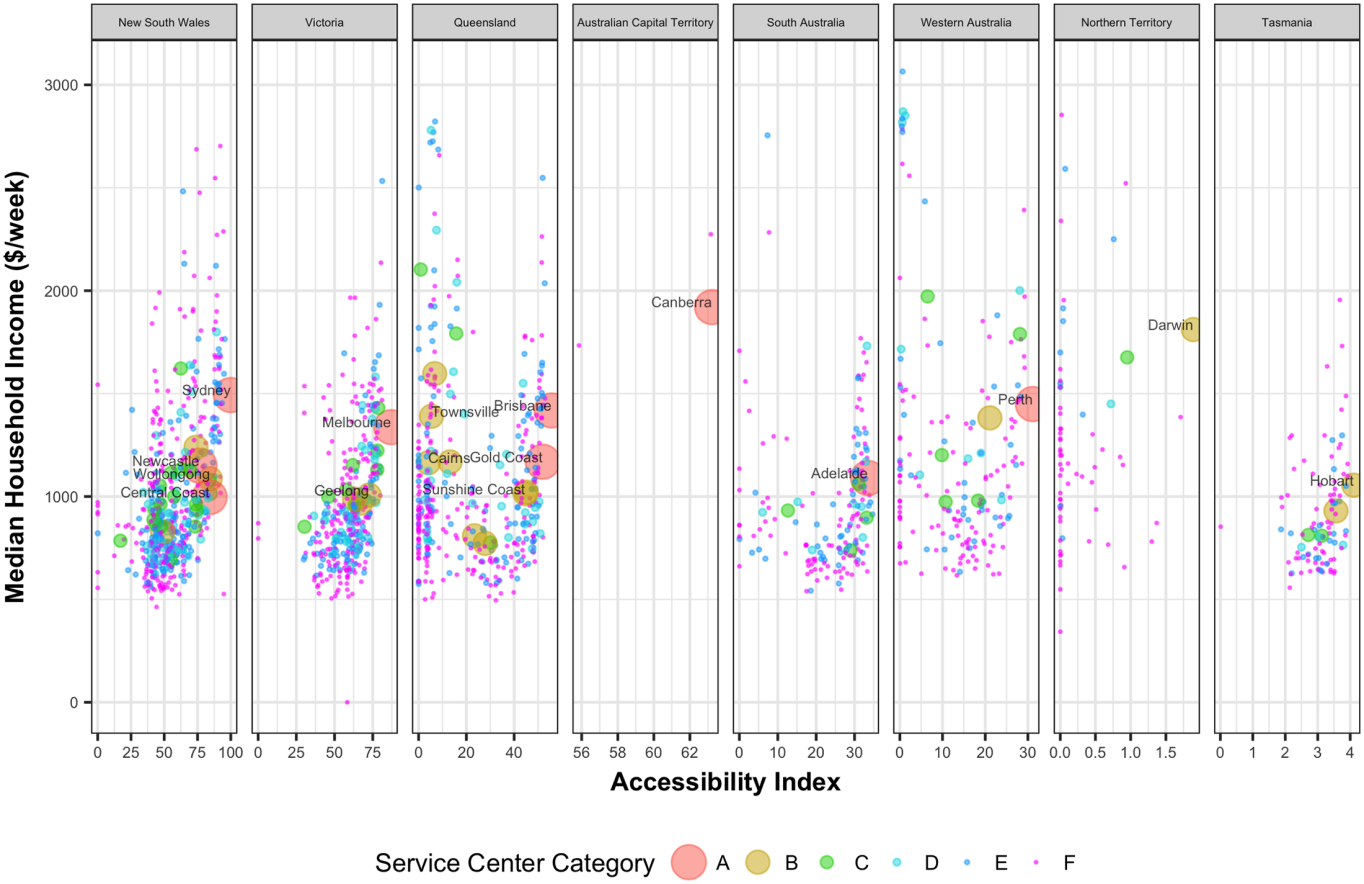
Median household weekly income of population against the corresponding normalized accessibility values of the UCLs.

Intuitively, the consequence of unequal access to services and opportunities will also be reflected in income distribution. But as we plot median household weekly income against the accessibility values in Fig 5, we observe that all of the major capital cities have similar median household income irrespective of their accessibility values. Darwin, which has the lowest accessibility value among the capital cities, has one of the highest median household weekly incomes in the country. However, majority of the UCLs belonging to service center categories C to F have lower income with decreasing accessibility values. The influence of accessibility on income is more prominent in state distribution. It shows that with decreasing accessibility, household median income also tends to decrease across all the states. There are some UCLs in Queensland and Western Australia with low accessibility but high median income; theses UCLs mostly represent the mining towns. Without a long-term investment and the development of a more diverse economic base, the communities in these UCLs could be severely impacted following the mining downturn leading to population migration from these regions which in turn can lead to further reduction in services and opportunities.

**Fig 6:**
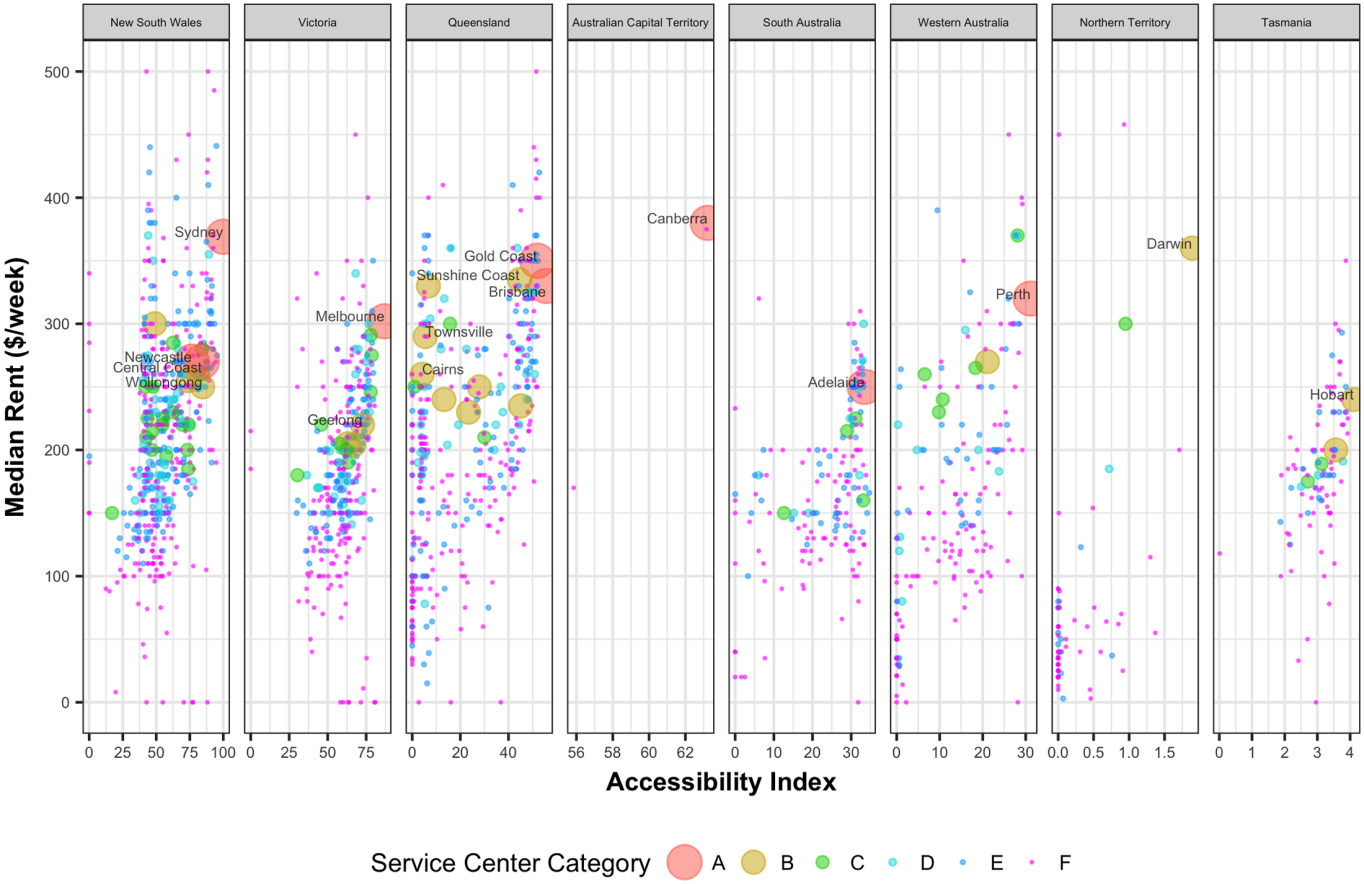
Median household weekly rent against the corresponding normalized accessibility values of the UCLs.

**Fig 7:**
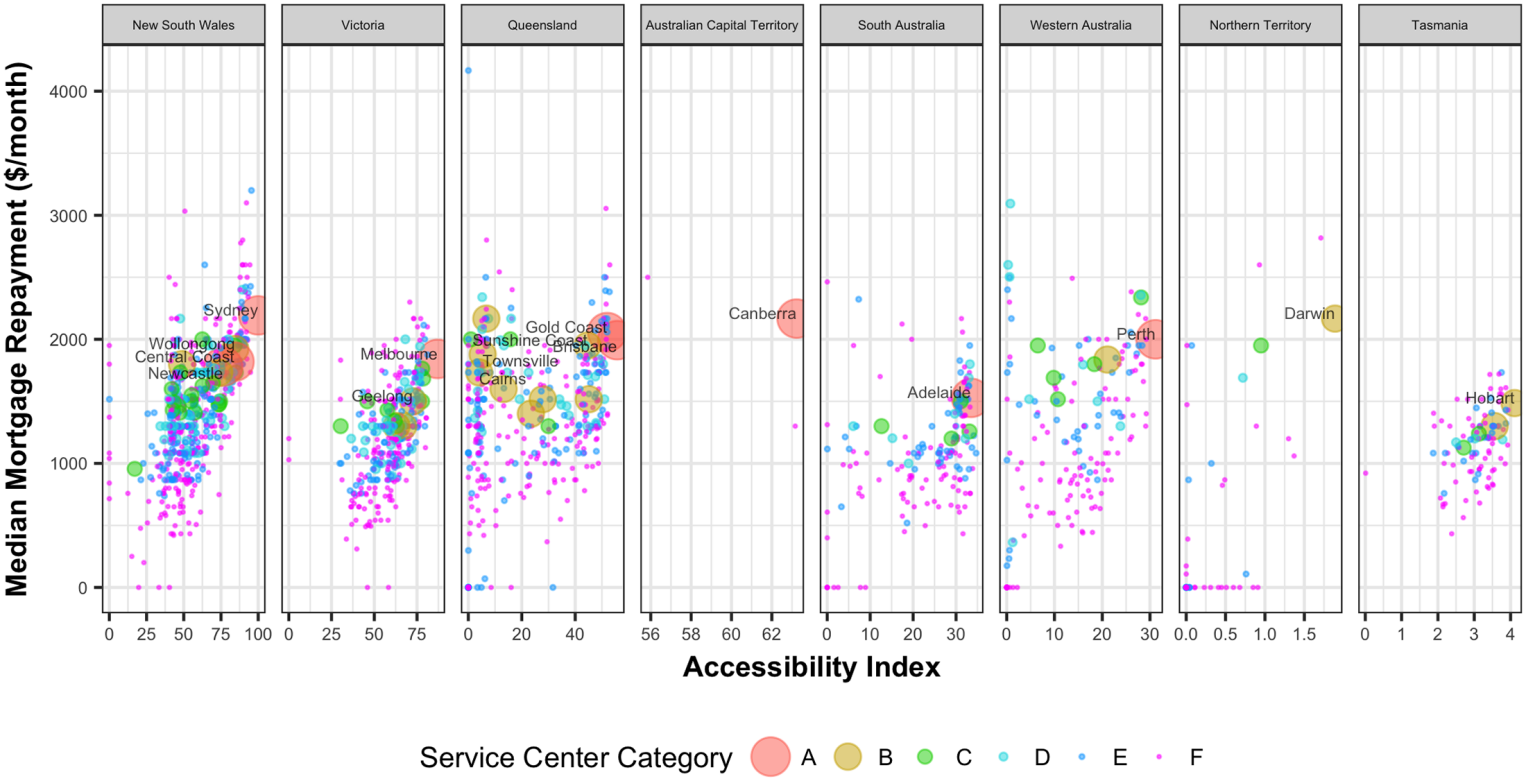
Median mortgage repayment against the corresponding normalized accessibility values of the UCLs.

A significant portion of household income is spent on rent or mortgage repayment. Fig 6 shows the median weekly rent in the UCLs against their accessibility values. With decreasing accessibility values housing rent tends to decrease except in the major capital cities. Fig 7 shows the median monthly mortgage repayment in the UCLs against their accessibility values. With decreasing accessibility values, mortgage payment tends to decrease except in the major capital cities. Similar to house rent, this is also consistent across all the states.

**Fig 8:**
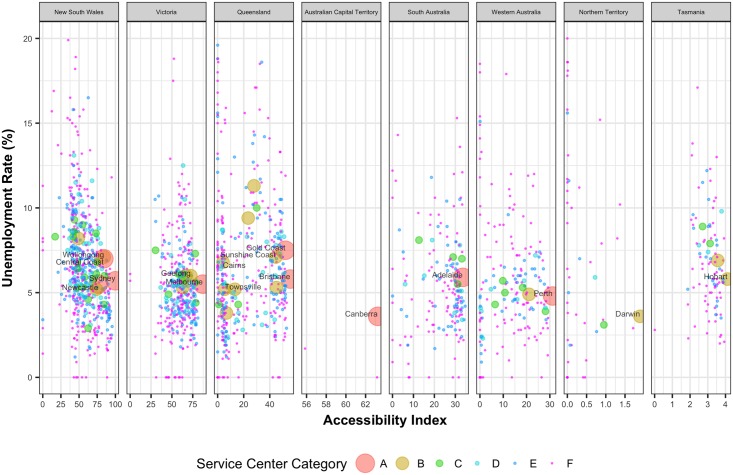
Unemployment rate of population against the corresponding normalized accessibility values of the UCLs.

Access to opportunities should have an important influence on unemployment rates. Fig 8 shows the unemployment rates of the UCLs against their corresponding accessibility values. In general, most of the major capital cities have lower unemployment rates. For instance, among the major cities Canberra and Darwin have the lowest unemployment rates. But for other UCLs, with decreasing accessibility values, unemployment rates tend to increase.

The Socio-Economic Indexes for Areas (SEIFA) set indicates the socio-economic conditions of an area created using census data. There are four SEIFA indexes available for analysis:

- The Index of Relative Socio-Economic Advantage and Disadvantage (IRSAD)
- The Index of Relative Socio-Economic Disadvantage (IRSD)
- The Index of Education and Occupation (IEO)
- The Index of Economic Resources (IER).

**Fig 9:**
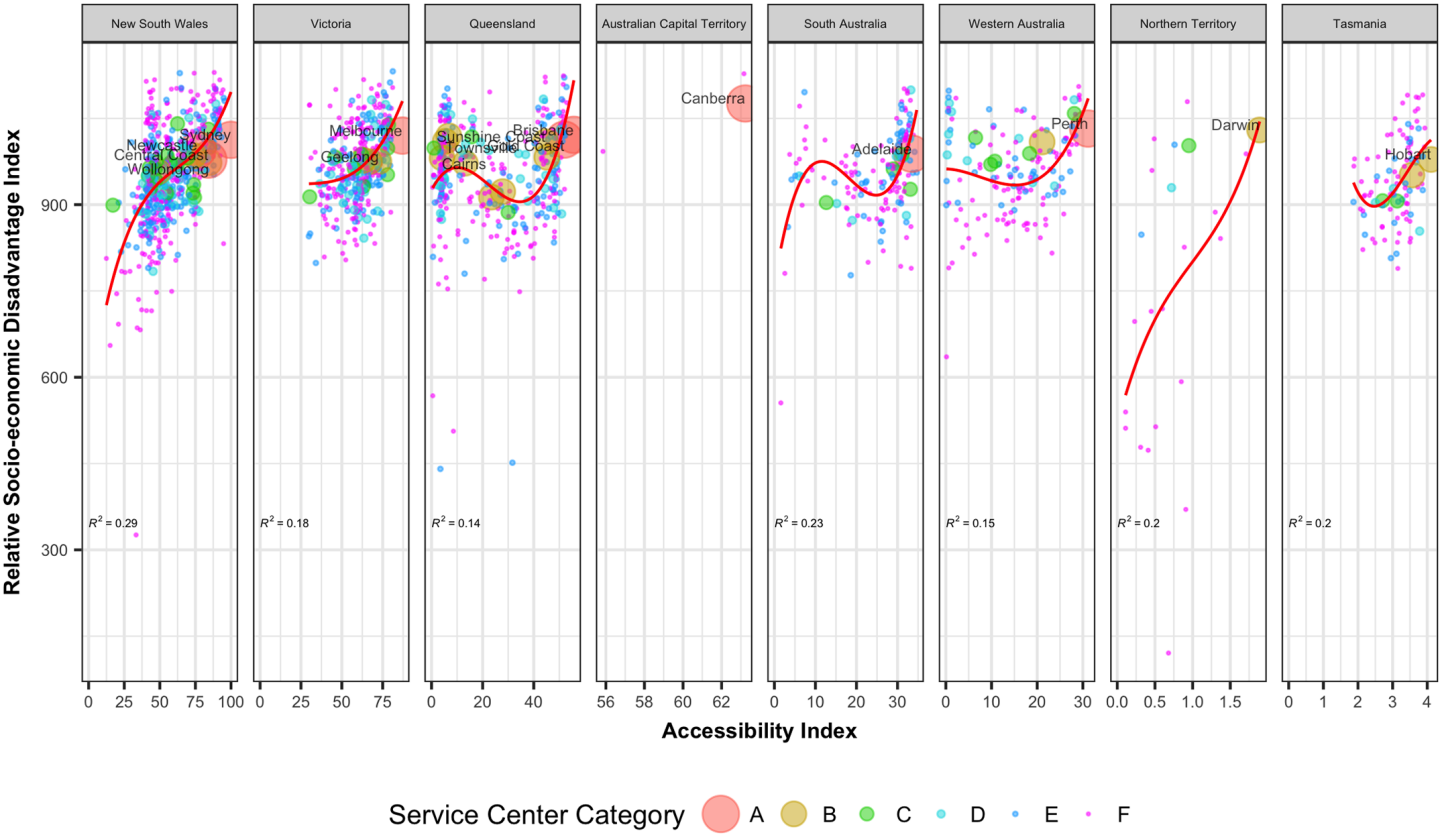
The relative index for socio-economic disadvantage of population against the corresponding normalized accessibility values of the UCLs (trends are fitted with an equation of the form *y* = *α* + *β*_1_*x + β*_2_*x*^2^ + *β*_3_*x*^3^ and R squared values of the fitted equations are reported).

Each index is a summary of a different subset of census variables and focuses on a different aspect of socio-economic advantage and disadvantage. We plot these 4 SEIFA indexes of the UCLs against their corresponding accessibility values. Fig 9 shows the Index of Relative Socio-Economic Disadvantage (IRSD) against the accessibility values of the UCLs. The IRSD summarizes the economic and social conditions of people and households within an area including only measures of relative disadvantage (24). A low value of this index indicates relatively greater disadvantage in an area including, for example, many households with low income, many people with no qualifications, or many people in low skill occupations. On the other hand, a high value of the index indicates a relative lack of disadvantage in an area including, for example, few households with low incomes, few people with no qualifications, and few people in low skilled occupations. There is a strong correlation between IRSD and accessibility values. With the decrease of accessibility to services and opportunities, the index for relative socio-economic disadvantages also reduces. Figures G-I in S1 File plot the remaining SEIFA indexes against the accessibility values. The pattern of decreasing socio-economic advantages, economic and education resources with decreasing accessibility values is consistent across all the figures.

## Discussion and implications

Our analysis of the relative accessibility of all the significant human settlements in Australia provides a better understanding of the hierarchical ranking among Australia’s cities and towns, and how these relate to socio-economic development factors. It shows the dominant position of its two largest cities, Sydney and Melbourne, across all performance indicators, compared to all the other cities, in support of the concept of *dragon kings* [27,28], like the role of London in the UK [22]. This hierarchical ranking based on accessibility values is closely related to the observed scaling relationships with city size [29,30]. Our approach, however, presents a different perspective to this question adding a new dimension of geographic distances. This hierarchical ranking is possibly the outcome of a complex process involving resource accumulation [31,32], income agglomeration in big cities [33], preferential attachment of migrating population [34] and high investment endowment to connect to big cities. This poses both challenges and opportunities even for a developed nation such as Australia. Because of the sparsity of the major cities and a continental land mass to cover, Australia has to build and maintain a massively large transport network compared to a small population the network serves. However, this also brings an enormous opportunity to serve the growing population demand by strategically locating or supporting new growth centers in the existing network through investments in infrastructure and government services. Such public policy process can be initially guided by the accessibility ranking presented herein.

In relating the accessibility values of the UCLs with their socio-economic characteristics, we observed the following:

- There is an increasing trend of accessibility values originating from the eastern coast cities, particularly, from Brisbane, Sydney, and Melbourne. A similar pattern is observed in Western Australia with Perth as the source of the trend.
- There is a strikingly unequal distribution of the access to opportunities in Australia. Unlike the south-eastern and the south-western regions, there such a trend is not observed in Northern Australia. Investing in road infrastructure in Northern Australia could start a similar trend of increasing accessibility from a major city center (such as Darwin) potentially leading to improvements in socio-economic development and feeding the growth pattern in the region. The assessment of such development options should, however, be undertaken balanced with their potential impacts on the environment and/or ecological services.
- Unequal access to opportunities has a strong association with socio-economic characteristics, particularly for the dispersed and remote areas. With decreasing accessibility, median age and unemployment rate tend to increase and median household income tends to decrease.

Our analysis also reveals that there is a strong presence of an *island effect* in Australia’s regional development. Regional cities/towns benefit from the services and opportunities available to the nearest major capital cities (Fig 3). In addition, most of the development starts from major capital cities (e.g., Sydney, Melbourne, Brisbane etc.) supported by medium or small urban centers. However the diffusion of growth [35,36] from one major capital city cannot reach farther because of distance (except in the Melbourne-Sydney-Brisbane growth corridor) and hence fails to create a continuum of development. It is likely that future development growths will also follow similar patterns starting from major capital cities. However, the major investment initiatives improving accessibility expecting growth and development in remote areas should focus more from a regional development perspective instead of concentrating to an isolated city or locality.

Access to opportunities has been found to be strongly associated with certain socio-economic characteristics of human settlements. Although such correlations were difficult to find in more developed regions such as Europe through empirical evidence [10], for Australia the evidence is more prominent. For instance, although major capital cities have lower median age due to the influx of young immigrant population, most small cities (category D-F) have significantly higher median age. While the aging populations have greater mobility and accessibility expectations and needs [37], current trend indicates that they remain or migrate to regions with less access to opportunities. This is perhaps linked with the higher housing costs of major cities in Australia and the limited financial capability of some of the elderly populations [38]. Our analysis also confirms some of the common characteristics [39] of the regional and remote areas in Australia including the lower income groups of people living in these areas; reduced access to services, and declining employment opportunities. Recent findings based on Australian cities also suggest that socio-economic inequality grows with city size [33].

Our analysis, however, has some limitations which can be addressed from several perspectives. The proposed definition of accessibility is based on population size which is assumed as a proxy to the level of services available to a city. This definition can be extended to consider specific dimensions of urban services including housing, health, education, financial, professional and recreational, among many others. A service-specific definition of accessibility will provide interesting insights on different dimensions of urban services. However, for a high-level strategic planning perspective a generic indicator such as the proposed accessibility index should be sufficient. To define settlements, we have used a particular geographic classification provided by ABS. A recent work [27] on urban scaling laws has found lack of consistency of scaling exponents across city definitions. It needs to be investigated if similar inconsistencies are also observed for accessibility based ranking of cities. The empirical evidence that regions with lower accessibility have lower quality of life answers an important question on the value of accessibility on socio-economic development. However, this finding is based on the observed correlations. Further analysis is needed to determine the underlying causes of socio-economic underperformance of lower ranked cities.

**Fig 10:**
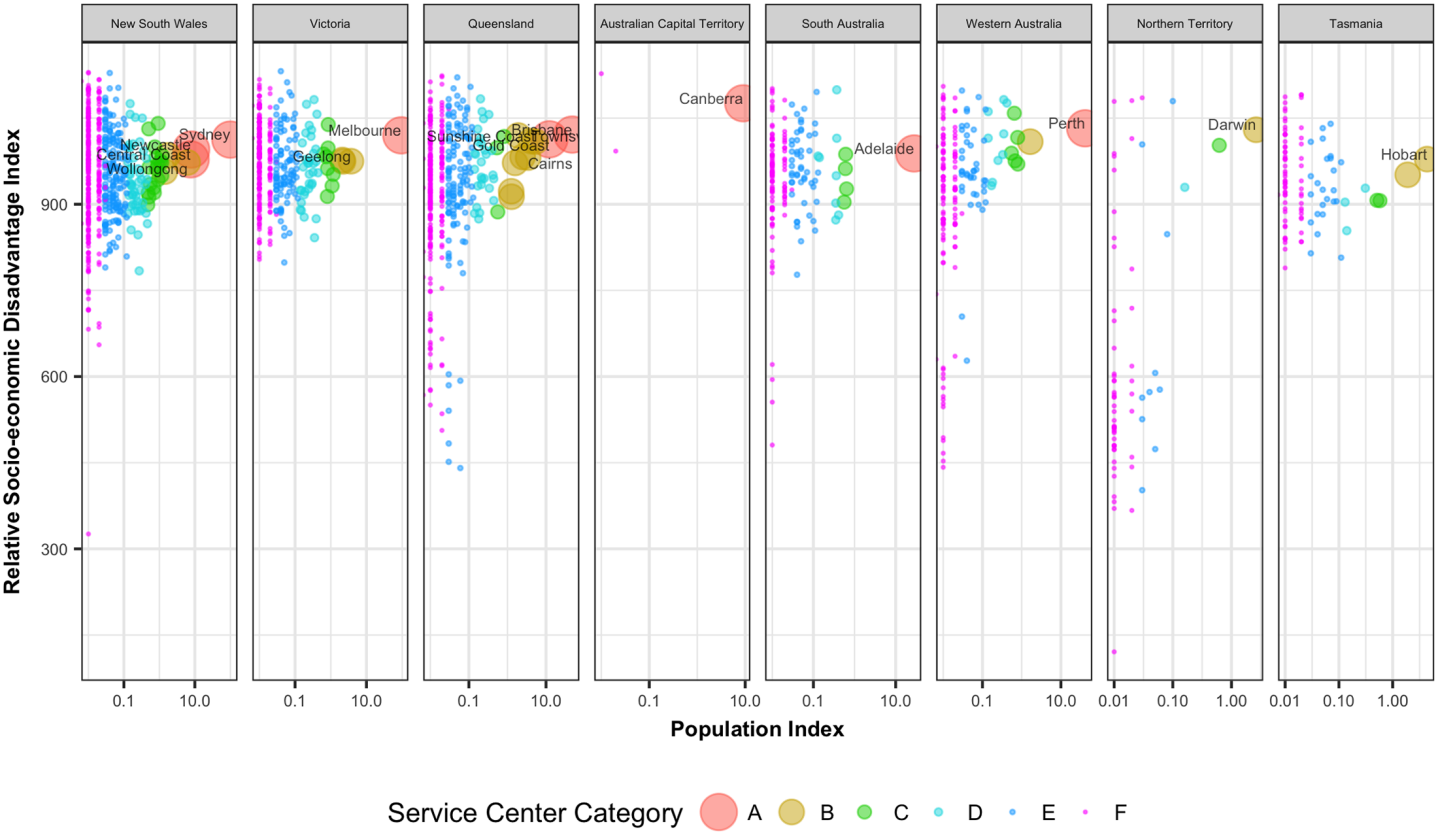
Relative socio-economic disadvantage index vs. normalized population-based index values of the UCLs (where $Normalized\;Population\;Index{\;}_{i}=\;\frac{Population_{i}}{Population_{Sydney}}\times 100$).

There are alternative computations of accessibility values. One possible alternative is to consider only the opportunities available in the origin UCL ignoring the opportunities from all other UCLs. This is nothing but a simple index based on population size only. A ranking based on a population-based index will be quite different than the one based on the accessibility index presented herein. For example, a UCL with a small population size near a major city will have a very high accessibility value because of the opportunities present in the nearby city. Whereas a population based indicator will give it a lower value because of a small population size. Fig 10 shows the correlation between socio-economic disadvantage against a simple population-based index whereas Fig 9 shows the correlation between the same variable against the proposed accessibility-based index. It is clear that, compared to population size alone, accessibility does a better job in explaining the variations in socio-economic disadvantages present in different regions.

Our study provides guidance to Australian policy makers about the potential impact(s) of investment (or investment options) on infrastructure, and/or in identifying what investment is needed to stimulate regional development (or specifically targeted communities/UCLs). This is particularly relevant to its stated vision of developing the communities, economies and businesses in Northern Australia. First, our results (Fig 1) especially confirm that this region is indeed one that is most in need of improving its accessibility infrastructure. Second, policy makers can identify what equivalent socio-economic level they would like to aim for in specified settlements in the region (based on more established or accessible UCL or communities elsewhere), in terms of the latter’s accessibility index and socio-economic profile. The impacts of targeted distribution of the government’s $5 billion infrastructure fund [16] could be maximized. The government can ensure that socio-economically disadvantaged people in specific communities have access to services and economic opportunities. Re-assessment of investment options will result in different ranking results, quantifying the improvements in accessibility index, and allowing a systematic prioritization process. It is hard to say whether a similar analysis in other parts of the world will yield similar benefits, and thus, this should be tested elsewhere [40].

## Supporting information

S1 File Data description and additional figures. (DOCX)

S2 File Accessibility index values. (CSV)

## References

[1] Reggiani A, Nijkamp P, Lanzi D. Transport resilience and vulnerability: The role of connectivity. Transp Res Part A Policy Pract. Elsevier Ltd; 2015;81: 4–15. doi: 10.1016/j.tra.2014.12.012

[2] Litman T TA. Accessibility A Dictionary of Transport Analysis. 2010 pp. 1–3.

[3] Condeço-Melhorado A, Reggiani A, Gutiérrez J. Accessibility and Spatial Interaction. Edward Elgar, Cheltenham; 2014.

[4] Handy SL, Niemeier DA. Measuring accessibility: an exploration of issues and alternatives. Environ Plan A. 1997;29: 1175–1194.

[5] Handy S. Regional versus local accessibility: Implications for nonwork travel. Univ Calif Transp Cent. 1993;

[6] Vickerman RW. Transport infrastructure and region building in the European Community. J Common Mark Stud. 1994;32: 1–24.

[7] Biehl D. The role of infrastructure in regional development. Infrastruct Reg Dev. 1991; 9–35.

[8] Hansen WG. How Accessibility Shapes Land Use. J Am Inst Plann. 1959;25: 73–76. doi: 10.1080/01944365908978307

[9] Demurger S. Infrastructure development and economic growth: an explanation for regional disparities in China? J Comp Econ. 2001;29: 95–117.

[10] Vickerman R, Spiekermann K, Wegener M. Accessibility and economic development in Europe. Reg Stud. 1999;33: 1–15.

[11] Gray I, Lawrence G. A future for regional Australia: Escaping global misfortune. Cambridge University Press; 2001.

[12] Department of Health and Aged Cared (DHAC). Measuring remoteness: Accessibility/remoteness index of Australia (ARIA) (Revised edition). Canberra: Department of Health and Aged Cared; 2001.

[13] Taylor MAP, Susilawati. Remoteness and accessibility in the vulnerability analysis of regional road networks. Transp Res Part A Policy Pract. Elsevier Ltd; 2012;46: 761–771. doi: 10.1016/j.tra.2012.02.008

[14] McKenzie FH. Attracting and retaining skilled and professional staff in remote locations of Australia. Rangel J. 2011;33: 353–363.

[15] Haslam McKenzie F. Fly-In Fly-Out: The Challenges of Transient Populations in Rural Landscapes In: Luck GW, Black R, Race D, editors. Demographic Change in Australia’s Rural Landscapes. Springer Netherlands; pp. 353–374.

[16] The Commonwealth of Australia. Our North, Our Future: White Paper on Developing Northern Australia [Internet]. 2015. https://northernaustralia.dpmc.gov.au/white-paper

[17] The Economist Intelligence Unit. Global liveability ranking. 2015.

[18] Smith MS. The “desert syndrome”—causally-linked factors that characterise outback Australia. Rangel J. Smith MS (reprint author), CSIRO, Sustainable Ecosyst, POB 284, Canberra, ACT 2601, Australia CSIRO, Sustainable Ecosyst, Canberra, ACT 2601, Australia mark.staffordsmith@csiro.au; 2008;30: 3–14. doi: 10.1071/RJ07063

[19] Fullerton B, Gillespie A. Transport and communications. Reg Impact Community Policies Eur. 1988; 88–110.

[20] Rietveld P, Nijkamp P. Transport and regional development. Eur Transp Econ. 1993; 130–151.

[21] Banister D, Berechman Y. Transport investment and the promotion of economic growth. J Transp Geogr. 2001;9: 209–218. doi: 10.1016/S0966-6923(01)00013-8

[22] United Nations. World Urbanization Prospects: The 2014 Revision. Department of Economic and Social Affairs, Population Division; 2015.

[23] Martín JC, Reggiani A. Recent Methodological Developments to Measure Spatial Interaction: Synthetic Accessibility Indices Applied to High-speed Train Investments. Transp Rev. 2007;27: 551–571. doi: 10.1080/01441640701322610

[24] Reggiani A. Accessibility, connectivity and resilience in complex networks Accessibility Analysis and Transport Planning: Challenges for Europe and North America. 2012 pp. 15–36.

[25] Taylor MAP, Susilawati. Remoteness and accessibility in the vulnerability analysis of regional road networks. Transp Res Part A Policy Pract. 2012;46: 761–771. http://dx.doi.org/10.1016/j.tra.2012.02.008

[26] Australian Bureau of Statistics. Technical Paper: Socio-Economic Indexes for Areas (SEIFA) 2011. 2013.

[27] Arcaute E, Hatna E, Ferguson P, Youn H, Johansson A, Batty M. Constructing cities, deconstructing scaling laws. J R Soc Interface. 2015;12: 20140745 doi: 10.1098/rsif.2014.0745 25411405 10.1098/rsif.2014.0745 PMC4277074

[28] Sornette D. Dragon-kings, black swans and the prediction of crises. Swiss Financ Inst Res Pap. 2009;

[29] Bettencourt LM a, Lobo J, Helbing D, Kühnert C, West GB. Growth, innovation, scaling, and the pace of life in cities. Proc Natl Acad Sci U S A. 2007;104: 7301–7306. doi: 10.1073/pnas.0610172104 17438298 10.1073/pnas.0610172104 PMC1852329

[30] Bettencourt LM a. The origins of scaling in cities. Science (80-). 2013;340: 1438–1441. doi: 10.1126/science.1235823 23788793 10.1126/science.1235823

[31] Friedmann J. The world city hypothesis. Dev Chang. 1986;17: 69–83.

[32] Behrens K, Robert-Nicoud F. Survival of the Fittest in Cities: Urbanisation and Inequality. Econ J. 2014;124: 1371–1400. doi: 10.1111/ecoj.12099

[33] Sarkar S, Phibbs P, Simpson R, Wasnik S. The scaling of income distribution in Australia: Possible relationships between urban allometry, city size, and economic inequality. Environ Plan B Plan Des. 2016; 265813516676488. doi: 10.1177/0265813516676488

[34] Barabási A-L, Albert R. Emergence of Scaling in Random Networks. Science (80-). 1999;286: 509–512. doi: 10.1126/science.286.5439.509 10.1126/science.286.5439.509 10521342

[35] Fotheringham AS, Batty M, Longley PA. Diffusion-limited aggregation and the fractal nature of urban growth. Pap Reg Sci. 1989;67: 55–69.

[36] Makse HA, Havlin S, Stanley HE. Modelling urban growth patterns. Nature. 1995;377: 608–612.

[37] Alsnih R, Hensher DA. The mobility and accessibility expectations of seniors in an aging population. Transp Res Part A Policy Pract. 2003;37: 903–916.

[38] Duncombe W, Robbins M, Wolf DA. Retire to where? A discrete choice model of residential location. Int J Popul Geogr. 2001;7: 281–293.

[39] National Rural Health Alliance Inc. A snapshot of poverty in rural and regional Australia. 2013.

[40] Brelsford C, Lobo J, Hand J, Bettencourt LMA. Heterogeneity and scale of sustainable development in cities. Proc Natl Acad Sci. 2017; 201606033 doi: 10.1073/pnas.1606033114 28461489 10.1073/pnas.1606033114 PMC5576773

