# Supplementary material for: Accessibility and socio-economic development of human settlements
Source: PLoS One. 2017 Jun 21;12(6):e0179620. doi: 10.1371/journal.pone.0179620 (PMC5479555; doi:10.1371/journal.pone.0179620)
Supplement: S1 File — (DOCX) [file pone.0179620.s001.docx]

**Data Description and Additional Figures**

# Data

Accessibility indices for each UCL were estimated using population size and major sealed roads network. This document describes the source of these datasets.

## Population

Population size for each UCL is derived from the place of usual residence (PURP) from Table B01 of the Basic Community Profile (1) where PURP refers to “the address at which a person lives or intends to live for six months or more”. (<http://www.abs.gov.au/websitedbs/censushome.nsf/home/statementspersonpurp?opendocument&navpos=450>).


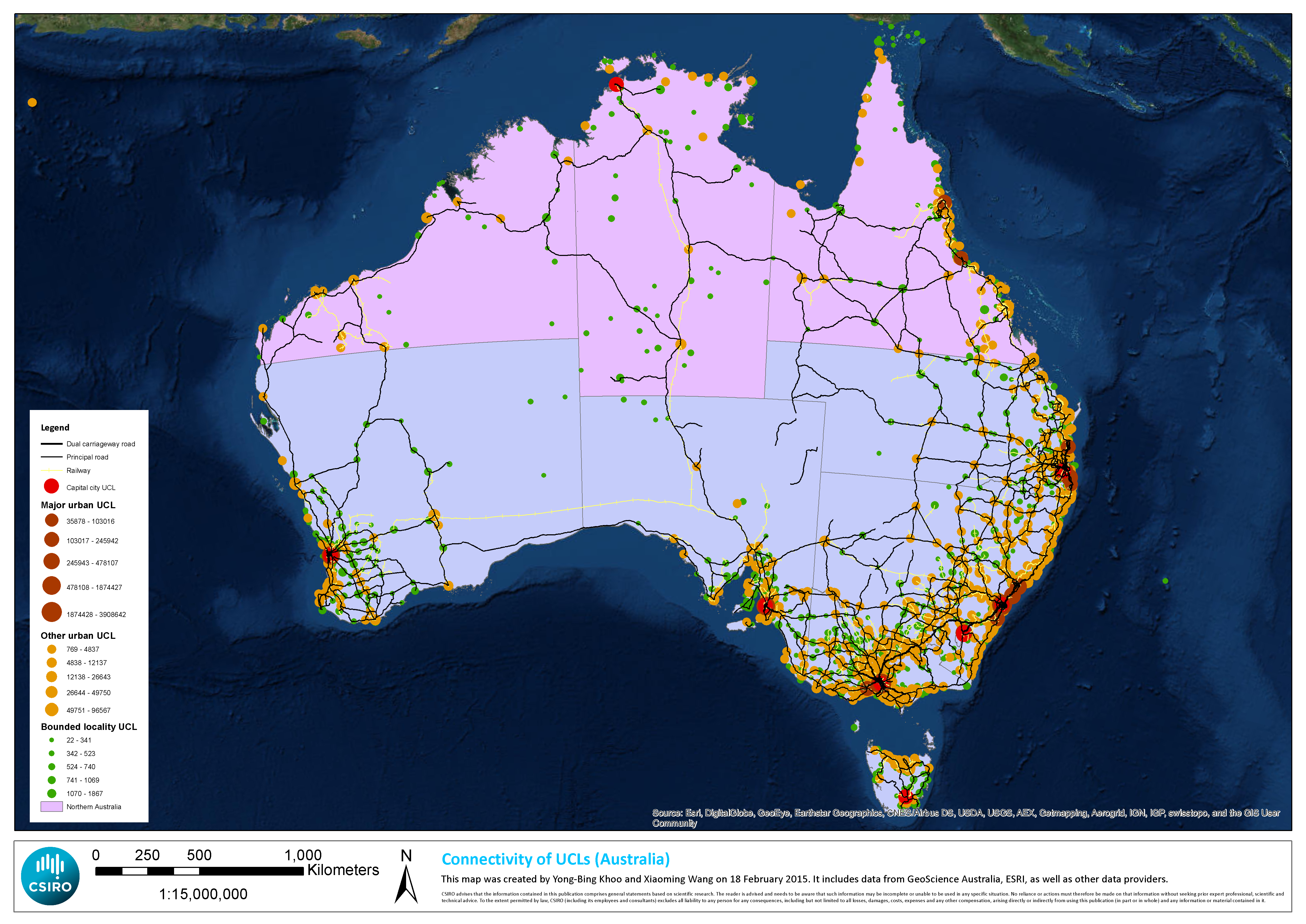


Fig. A Map of UCL place of usual residence population count and main roads

The 2011 UCL digital boundaries data in ESRI shapefile format can be downloaded from:

<http://abs.gov.au/AUSSTATS/subscriber.nsf/log?openagent&1270055004_ucl_2011_aust_shape.zip&1270.0.55.004&Data%20Cubes&52B516A3309048CBCA257A980013A96D&0&July%202011&16.10.2012&Latest>

## Road network

The road network dataset (Fig. A) used in this project is made up of all major sealed roads retrieved from Geoscience Australia’s GeoData Topo 250K Series 3 (<http://www.ga.gov.au/metadata-gateway/metadata/record/gcat_a05f7892-ecbd-7506-e044-00144fdd4fa6/GEODATA+TOPO+250K+Series+3+%28Packaged+-+Shape+file+format%29>). The dataset was tested by comparing the shortest distances derived using the dataset with the distances estimated by Google Map (Table 1). All tests passed within 5% of the expected distances.

Table 1 Expected shortest distances between UCLs computed from Google Map

| Source UCL | Destination UCL | Expected distance (km) |
| --- | --- | --- |
| Perth | Darwin | 4028 |
| Perth | PortHedland | 1649 |
| PortHedland | Darwin | 2414 |
| Perth | Brisbane | 4341 |
| Perth | Sydney | 3938 |
| Normanton | Darwin | 2100 |
| Melbourne | Brisbane | 1682 |
| Melbourne | Perth | 3420 |
| Melbourne | Adelaide | 725 |
| Melbourne | Darwin | 3752 |
| Melbourne | Sydney | 878 |
| Melbourne | Canberra | 663 |
| Melbourne | Dubbo | 832 |
| Dubbo | Brisbane | 851 |
| Dubbo | Moree | 378 |
| Moree | Goondiwindi | 125 |
| Moree | Boggabilla | 115 |
| Dubbo | Goondiwindi | 503 |

We have considered only sealed roads for computing distances, since unsealed roads have limited functions as compared to the sealed roads for receiving goods and services, generating opportunities broadly implied in this study. We believe that considering unsealed roads will assign accessibility values to very remote areas that are not comparable with other regions.

## Socio-Economic Indexes for Areas (SEIFA) Data

The SEIFA indexes data were collected from Australian Bureau of Statistics (ABS) website (<http://www.abs.gov.au/AUSSTATS/abs@.nsf/DetailsPage/2033.0.55.0012011?OpenDocument>).

The details of the indexes, variables used, and their uses can be found at the explanations provided by ABS (<http://www.abs.gov.au/AUSSTATS/abs@.nsf/Lookup/2033.0.55.001Explanatory%20Notes12011?OpenDocument>).

The SEIFA indexes used for our analysis were available at Statistical Level 1 (SA1), a geographical classification defined by ABS. Indexes for the UCLs are calculated by aggregating the relevant SA1 index scores, using the SA1 population size for weighting (2).


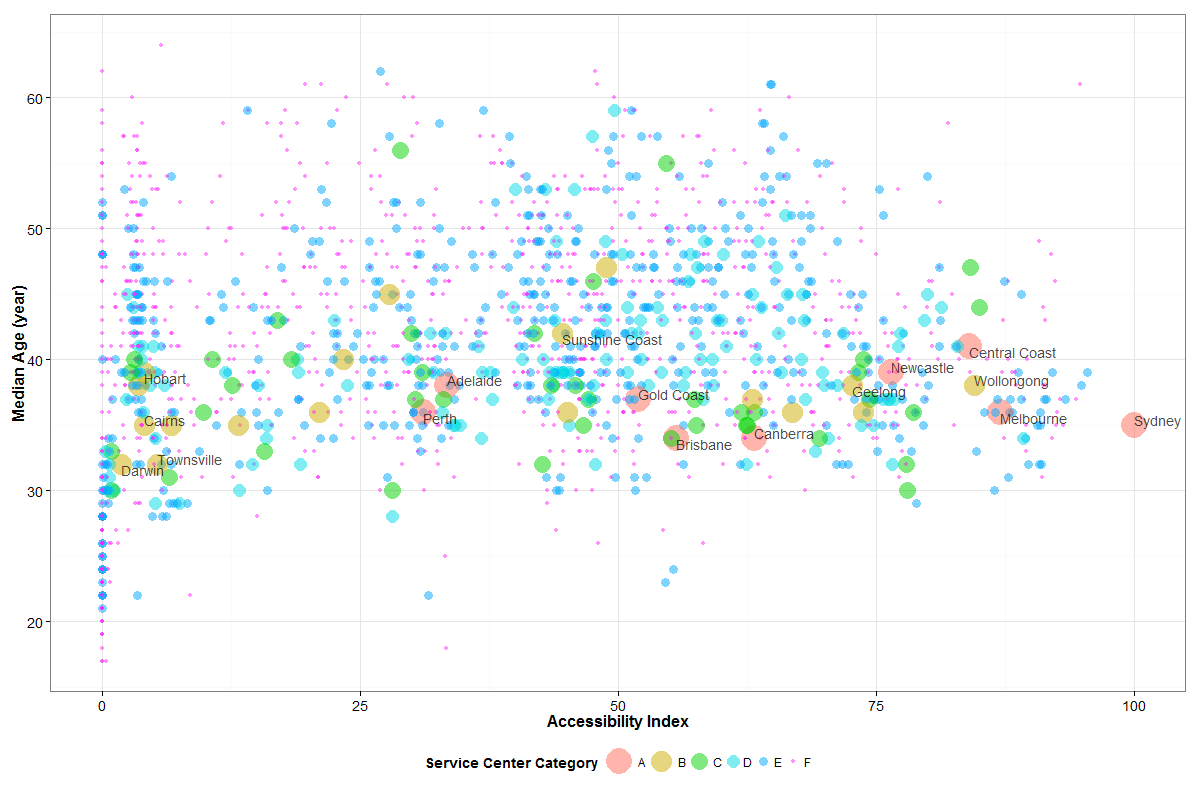


Fig. B Median age of the population against the accessibility values of the UCLs


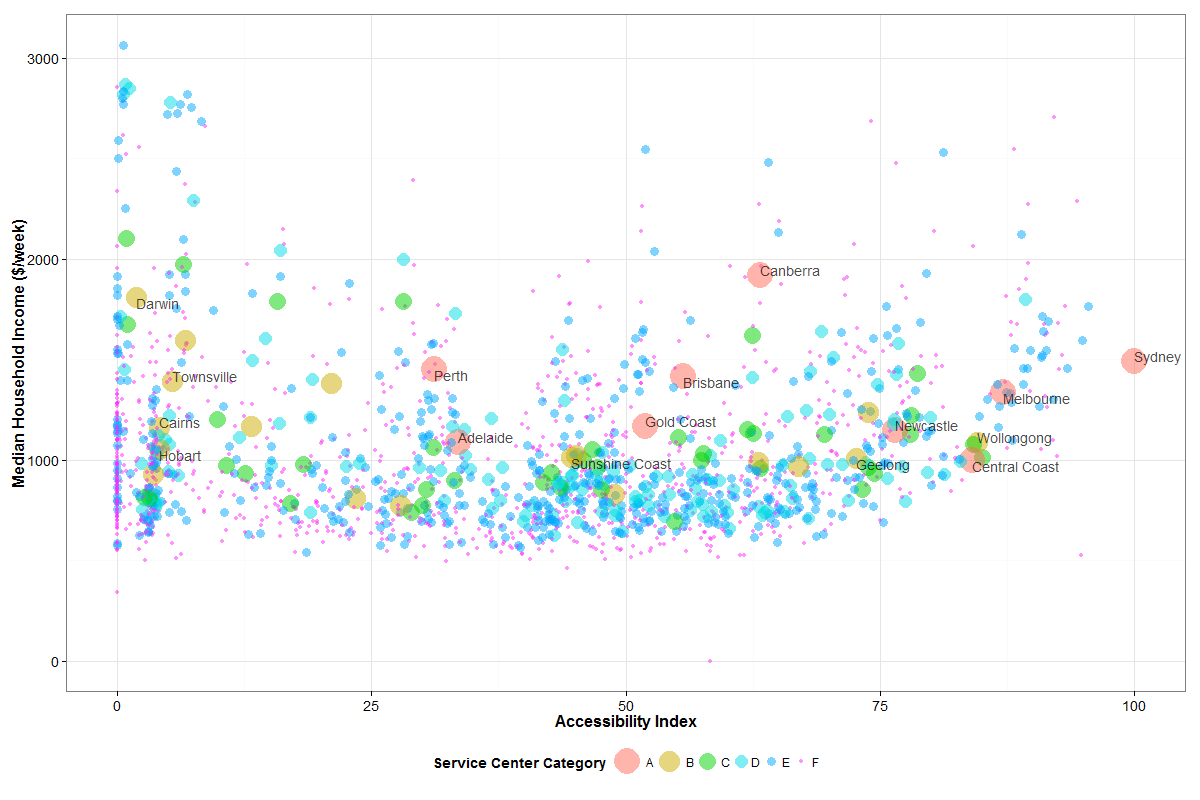


Fig. C Median household weekly income of the population against the accessibility values of the UCLs


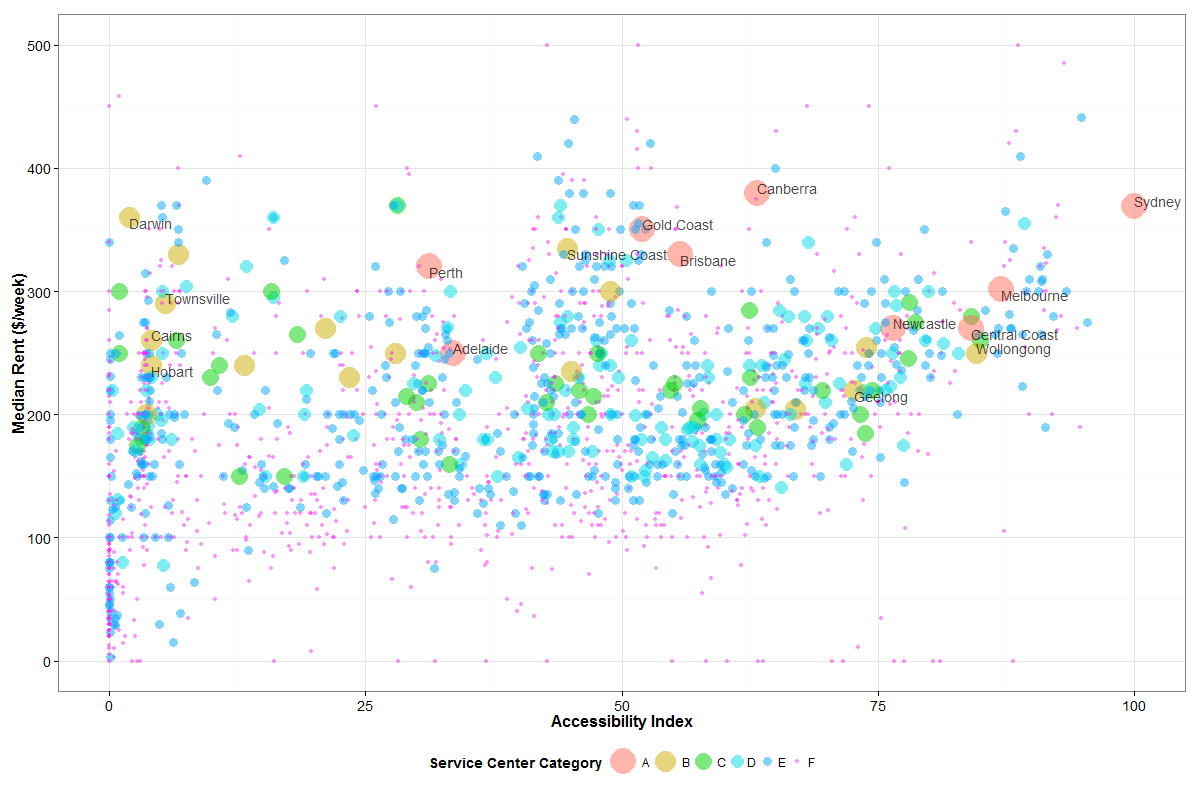


Fig. D Median household weekly rent against the accessibility values of the UCLs

##
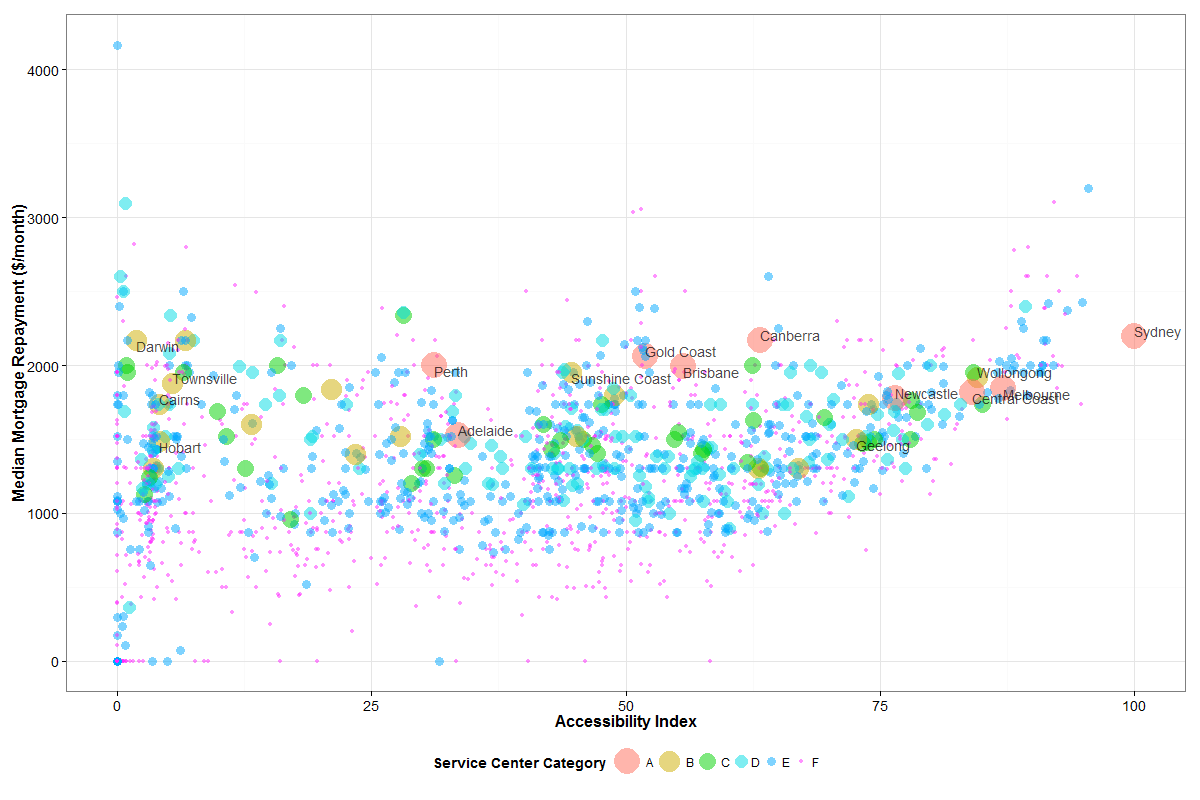


Fig. E Median mortgage repayment against the accessibility values of the UCLs


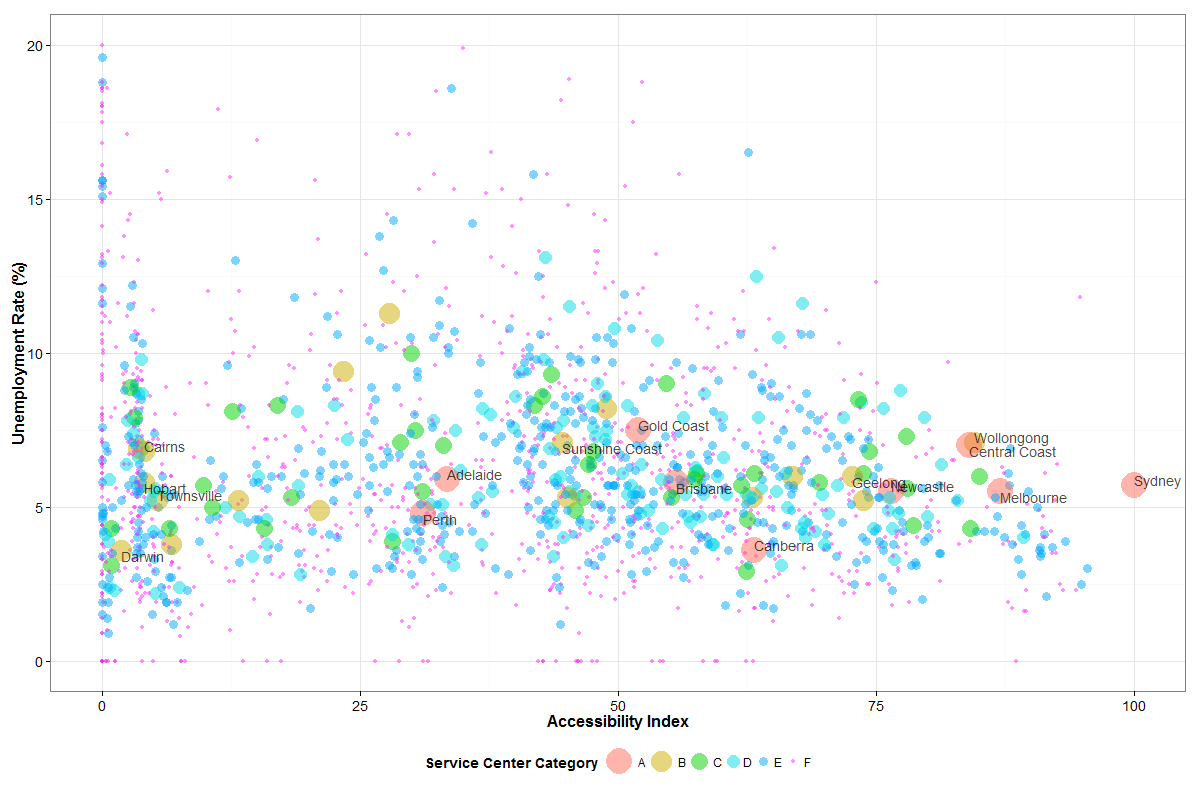


Fig. F Unemployment rate against the accessibility values of the UCLs

**
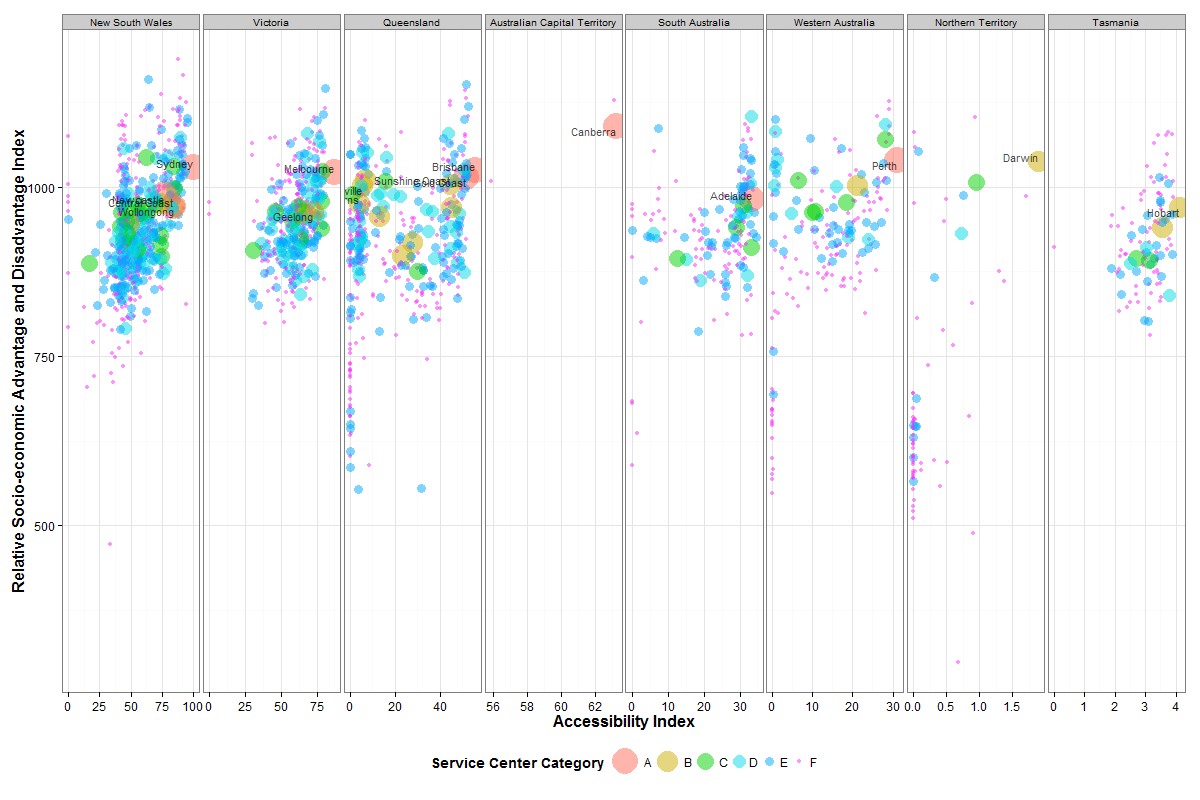
**

Fig. G The relative index for socio-economic advantage and disadvantage against the accessibility values of the UCLs. A low score indicates relatively greater disadvantage and a lack of advantage in an area including many households with low incomes, or many people in unskilled occupations and few households with high incomes, or few people in skilled occupations. A high score indicates a relative lack of disadvantage and greater advantage in an area including, for example, many households with high incomes, or many people in skilled occupations and few households with low incomes, or few people in unskilled occupations (2).

**
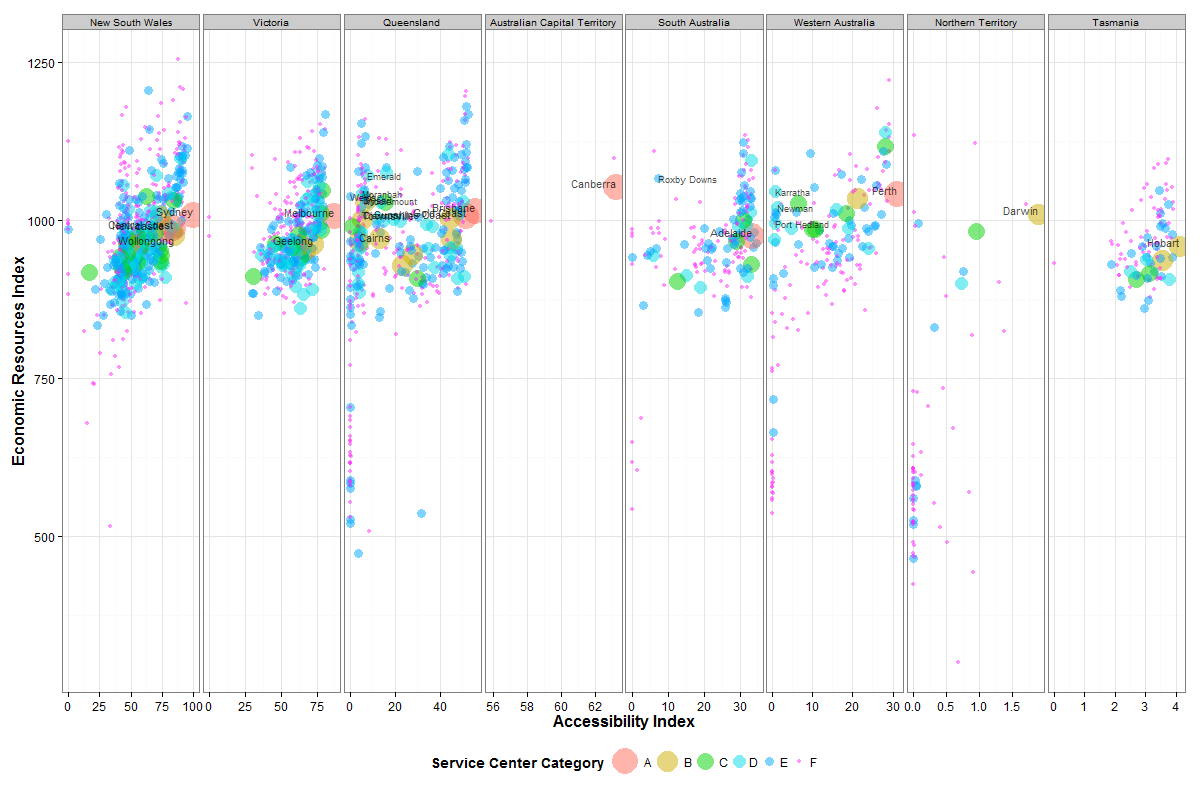
**Fig. H The index for economic resources against the accessibility values of the UCLs. A low score of the index indicates a relative lack of access to economic resources in an area including many households with low income, or many households paying low rent and few households with high income, or few owned homes. A high score indicates relatively greater access to economic resources in an area including many households with high income, or many owned homes and few low income households, or few households paying low rent (2).


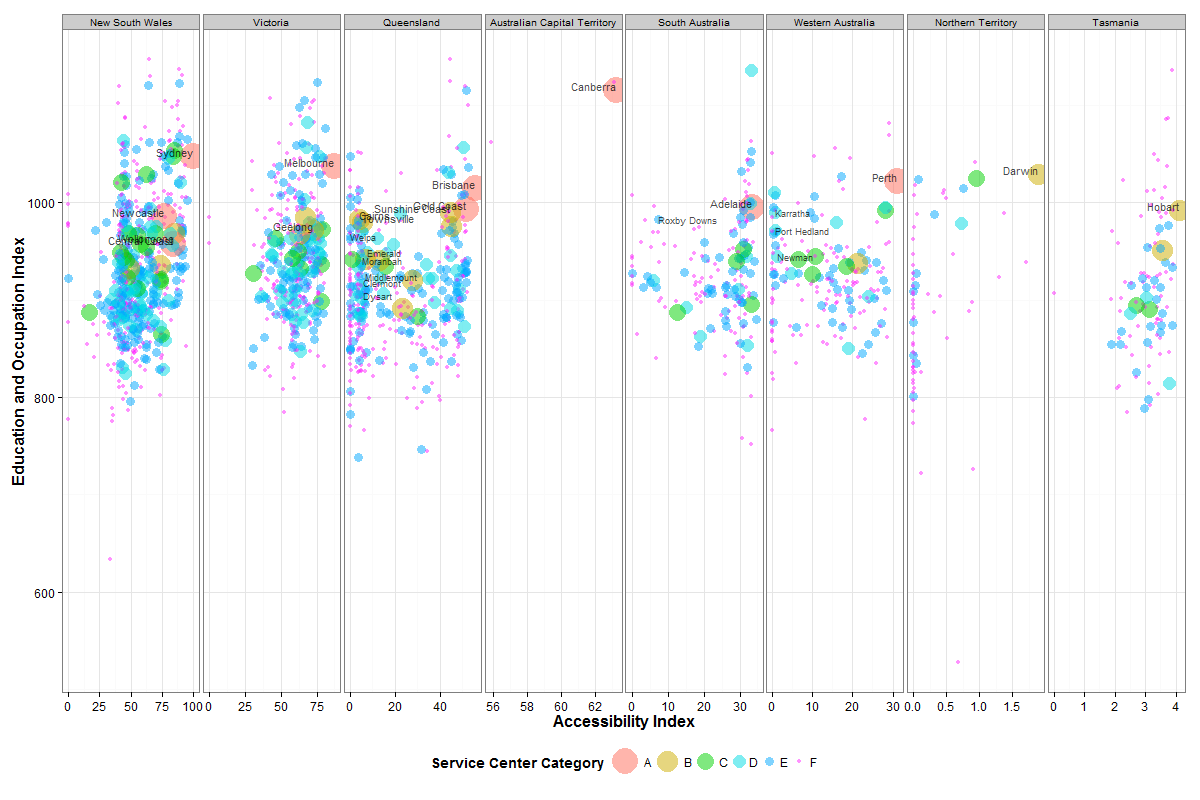


Fig. I The index for education and occupation against the accessibility values of the UCLs. A low score of the index indicates relatively lower education and occupation status of people in the area including many people without qualifications, or many people in low skilled occupations or many people unemployed and few people with a high level of qualifications or in highly skilled occupations. A high score indicates relatively higher education and occupation status of people in the area including many people with higher education qualifications or many people in highly skilled occupations and few people without qualifications or few people in low skilled occupations (2).

References

1. ABS (2011) Information Paper: Census of Population and Housing, Appendix 22: Basic Community Profile – List of Tables. .

2. Australian Bureau of Statistics (2013) Technical Paper: Socio-Economic Indexes for Areas (SEIFA) 2011.
